# Safety culture and perception of warning signs of chemical hazards among hospital cleaning workers: a cross-sectional study

**DOI:** 10.1186/s12889-023-15726-4

**Published:** 2023-05-04

**Authors:** Younes Mehrifar, Soleiman Ramezanifar, Parvaneh Khazaei, Afsane Azimian, Elahe khadiv, Ozra Dargahi-Gharehbagh, Ali Salehi Sahlabadi

**Affiliations:** 1 Department of Occupational Health and Safety, Student Research Committee, School of Public Health, Shahid Beheshti University of Medical Sciences, Tehran, Iran; 2 Department of Occupational Health and Safety, School of Public Health and Safety, Shahid Beheshti University of Medical Sciences, Tehran, Iran; 3 Environmental and Occupational Hazards Control Research Center, Department of Occupational Health and Safety at Work, School of Public Health and Safety, Shahid Beheshti University of Medical Sciences, Tehran, Iran

**Keywords:** Chemical safety, Hospital housekeeping, Health personnel, Hazard surveillance program, Perception, Product labeling, Safety culture

## Abstract

**Background:**

Due to the type of activities and the long-term exposure to chemicals, hospital cleaning workers require the necessary knowledge about the chemicals used and proper safety culture. This study aimed to evaluate the safety culture and perception of hospital cleaning workers' warning signs of chemical hazards.

**Methods:**

This cross-sectional study was conducted in 2022 with the participation of 68 cleaning workers with the mean age ± (SD) and work experience ± (SD) of 36.19 ± (7.619) and 9.21 ± (5.462), respectively, in four selected Tehran hospitals in Iran. After ensuring the confidentiality of the received information and completing the demographic information checklist, each participant completed Global Harmonization System (GHS) sign perception and the safety culture questionnaires in this survey. Data were analyzed using regression and Pearson correlation tests.

**Results:**

This study showed that the participant's correct perception in nine cases (81.8%) of presented GHS signs was lower than the ANSI Z535.3 standard. Among the investigated signs, "Flammable substances" and "Harmful to the environment" signs had the highest, and "Skin irritant" signs had the lowest correct perception. In addition, it was found that 55 people (80.9%) had an overall positive attitude toward the safety culture. The levels of "Work environment" (83.8%) and "Information exchange" (76.5%) had the highest and lowest positive scores for safety culture. Furthermore, there is a direct and significant relationship between the overall score of safety culture and the overall perception of the symptoms of GHS (CC = 0.313, *P* = 0.009).

**Conclusion:**

According to the obtained results, it is recommended to take the necessary measures to increase the employees' perception of the signs of chemical substances and improve their safety culture.

## Introduction

The emergence of new technologies, the use of chemicals, and the properties of some solvents have caused significant changes in the use of materials in processes and the type of activities in the workplace. According to their usage and importance, chemicals are used in industrial and non-industrial environments and small and large workshops [1, 2]. Considering the health risks of employees' exposure to chemical substances, it is necessary to have a minimum perception and awareness of the risks of using and being exposed to these substances in related occupational groups [3].

In the meantime, cleaning workers are one of the users who, due to the nature of their job (cleaning the surfaces and work environment from many types of pollution), are among the jobs exposed to the risks of chemical substances [4]. It is estimated that each cleaning worker uses about 110 kg of hazardous chemicals per year, depending on the nature of his job [5]. Many of these hazardous chemicals have corrosiveness, irritation, flammability, and oxidation properties [5–7]. They can be considered to be the cause of some respiratory (asthma, vaginitis), skin (dermatitis), fertility, and cardiovascular disorders [5, 8]. According to some studies, exposure to these chemical substances has caused hospital cleaning workers to suffer from occupational diseases such as occupational asthma and occupational contact dermatitis [9–11].

Furthermore, the unprincipled and incorrect use of these substances and not paying attention to chemical warning signs, in the long run, can cause a decrease in people's performance and ability, a decrease in their quality of life, and an increase in accidents in the workplace [12, 13]. Based on the studies of recent years in Iran, 9,886 accidents caused by work have been registered, of which 64 cases were due to exposure to dangerous chemicals [8].

In this regard, the Occupational Safety and Health Administration (OSHA) first created the Hazard Communication Standard (HCS). It then obliged employers to know the hazardous chemicals in the workplace and provide information about the dangers of these substances to employees [14]. In addition, in the last two decades, World Health Organization (WHO) and the International Labor Organization (ILO) have also tried to carry out activities in the field of chemical safety by taking measures such as the establishment of the Inter-Agency Program for the Correct Management of Chemicals (IOMC), the Strategic Approach to the International Management of Chemicals (SAICM), and the Global Harmonized System of Classification and Labeling of Chemicals (GHS) [8, 15, 16].

It is worth mentioning that the GHS has been proposed as an effective tool to increase users' perception of the nature of chemicals and the extent of chemical hazards. The goal of creating GHS is to provide an appropriate classification for chemicals according to their potential hazards and to convey essential information about these chemicals to users through standardized pictograms, signal words, hazard statements, and precautionary statements in labels and safety data sheets [7, 17]. Various criteria have been examined for the comprehensibility of chemical hazard pictograms. For example, using the ANSI Z535 and ISO 3864 standards, the acceptability of a symbol can be checked [18]. According to ANSI Z535 and ISO 3864 standards, signs and symbols must be understood by at least 85% and 67% of people, respectively [19].

Despite providing understandable labels and signs on chemicals, this information is only valuable when considered by the user. A recent study on household chemical products found that only 1.8% of participants stated that they always read the information on chemical containers [20]. It should be noted that if the work environment is safe, but the employees do not have the proper health knowledge, attitude, and behavior, most of the occupational safety and health measures and programs will fail [21]. Accident statistics show that humans are the main factor in most industrial accidents, and it is impossible to institutionalize safe behaviors in industries based only on technical engineering measures and establishing safety rules and regulations. Thus, by creating a positive and influential safety culture, people can be aware of existing risks and reduce accidents in the workplace [22, 23].

Safety culture is another important issue in preventing accidents and increasing people's positive attitude regarding occupational health issues [24, 25]. The term safety culture became popular after the Chernobyl nuclear power plant accident (Russia, 1986) [26]. Safety culture is considered a subset of the general culture of an organization, which includes values, methods, perception and awareness, competencies, and individual and collective behavior patterns that determine the commitments, how and the extent of management's performance towards health and safety of the organization [22]. Since compliance with safety will reduce errors and improve overall service quality, safety is considered a turning point for high-risk organizations such as hospitals [27–29]. Also, filling the wide gaps in organizations requires a safety culture [27].

The safety culture among different hospital departments has a significant difference [29]. A regular and correct assessment of the safety culture significantly reduces the occurrence of many foreseeable events and incidents that can impose a heavy financial burden on healthcare units. The safety culture reveals the strengths and weaknesses of the safety system and provides the basis for improving and changing the organization. In addition, safety culture evaluation will be a reliable measure to compare organizations and hospitals [30, 31].

**Fig.1:**
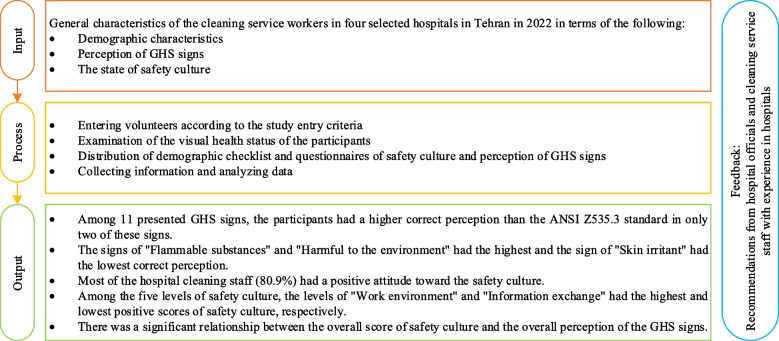
Research paradigm of the study

Hence, measuring the safety culture and the level of employees' perception of chemical signs in many jobs is highly recommended, according to the mentioned cases. One of these jobs is hospital cleaning workers, who spend most of their time with chemicals and disinfectants. According to the nature of their job, these employees should have a high safety culture and a correct perception of the signs of the chemicals they use. Therefore, due to the lack of research in this field (according to the investigations carried out by the research group) and the requirement to investigate this issue in this occupational group, this research was conducted to investigate the safety culture and the perception of hospital service employees about the warning signs of chemical hazards. Figure 1 shows the research framework used in the study.

## Methods

### Study design

This cross-sectional study was conducted with the participation of hospital service staff in Tehran (four hospitals: A, B, C and D) in August 2022. The Census method was used to estimate the sample size. For this purpose, after making the necessary arrangements with the hospital officials, a complete list of hospital cleaning staff consisting of 84 people was provided to the examiners. After applying the entry criteria (having at least one year of work experience, not having a second or more job, not having vision problems and having the same income range), it was determined that 79 people were eligible. In the next step, these employees were asked to participate in this research as volunteers by attending the hospitals, explaining the research objectives, and ensuring the confidentiality of the information received from the subjects. Employees who did not want to participate in this research (*n* = 9) and those who did not complete the questionnaires thoroughly (*n* = 2) were excluded. In general, 68 people were examined in this study.

After the staff's agreement and with the relevant officials' cooperation, the study participants were evaluated and ensured the absence of color blindness using the Ishihara test. One checklist and two questionnaires were collected, including the demographic information checklist, the GHS sign perception questionnaire, and the safety culture questionnaire. The demographic information checklist included information such as age, gender, education level, work experience, etc. It should be mentioned that in order to maintain the confidentiality of the information, each person was assigned a code to be used instead of their name. Also, after collecting the information, the completed questionnaires and their information were not given to anyone and were kept with the examiners.

### Assessment of perception of GHS signs

**Fig. 2:**
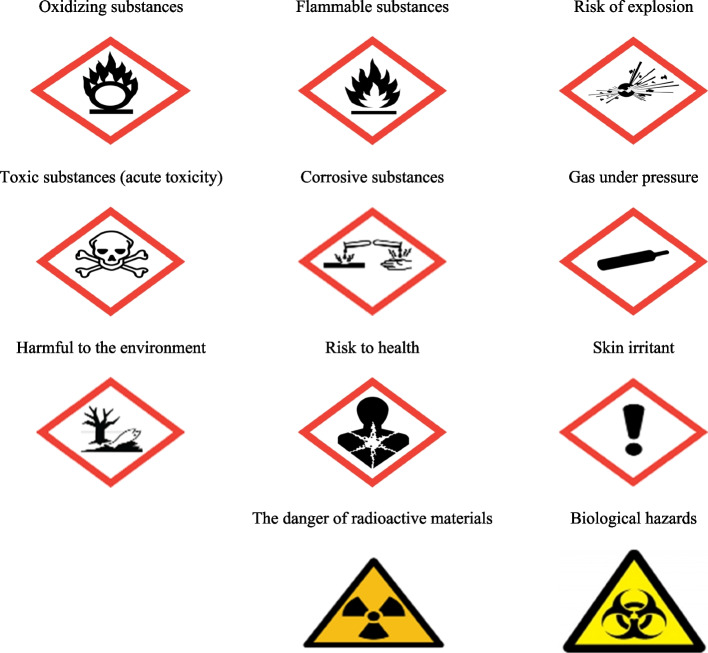
Pictograms used in the present study

The questionnaire for assessing participants' perception of GHS signs included 11 color pictograms along with their meanings, shown in Fig. 2. In this section, pictograms were presented with explanations of what the pictograms represented. In addition, to better understand the concepts of some pictograms, Hazard (H) statements were also used next to them [32]. For example, H315 was applied for the "Skin irritant" pictogram.

Then, employees were asked to answer their level of familiarity with these pictograms and concepts written next to them by choosing one of the "Correct", "Incorrect" and "I do not know" options. A score of one was given for the "Correct" answer, and a score of zero was given for the "Incorrect" answer and "I do not know".

### Safety culture assessment

The safety culture questionnaire was used to evaluate the safety culture of hospital employees. This questionnaire was designed and validated by Shekhari [22]. This tool has 40 questions in five levels "Education", "Work environment", "Information exchange", "Management commitment" and "Priority to safety". Each level has eight questions. The reliability of the present questionnaire was determined by the internal consistency method with Cronbach's alpha coefficient equal to 0.95. Questionnaire questions were graded according to a five-point Likert scale. Equation 1 is also used to evaluate the overall safety culture score and the safety culture score in each level [33].

1 $$\upmu =\frac{5k+k}{2}$$

Where:

 K: Total number of questions, and µ: Safety culture score.

According to the total number of questions of the questionnaire (K = 40), the overall score of positive safety culture is higher than 120, and the overall score of negative safety culture is lower than 120. In addition, in each of the safety culture levels, according to the total number of questions (K = 8), the positive safety culture score is higher than 24, and the negative safety culture score is less than 24.

### Statistical analysis of data

The collected data were analyzed by SPSS software version 24. Mean and Standard Deviation (SD) were used to express descriptive statistics. In addition, regression and Pearson correlation tests were used to express the analytical statistics and correlation of two variables, and a significance level of 5% was considered.

## Results

After distributing 70 questionnaires among cleaning workers working in selected hospitals, 68 questionnaires were completed and collected. According to the information received from the questionnaires, the mean age ± (SD) and work experience ± (SD) of the participants were 36.19 ± (7.619) and 9.21 ± (5.462), respectively. Most participants are male, married, have a diploma, and work in hospital A. All the participants were visually healthy according to the Ishihara test, and all used detergents. 10.3% of people did not receive special training on detergent use. The demographic information of the participants is presented in Table 1.

**Table 1:** Demographic characteristics of the participants of this study (*n* = 68, August 2022, Tehran)

Characteristic	Number (%)
**Gender**	
Female	26 (38.2%)
Male	42 (61.8%)
**Age (Years)**	
< 30 Years	19 (27.9%)
30–40 Years	30 (44.2%)
> 40 Years	19 (27.9%)
**Work experience (Years)**	
< 5 Years	21 (30.9%)
5–10 Years	20 (29.4%)
> 10 Years	27 (39.7%)
**Marital status**	
Married	44 (64.7%)
Single	24 (35.3%)
**Level of education**	
Elementary degree	7 (10.3%)
Middle degree	16 (23.5%)
Diploma degree	33 (48.5%)
Associate degree	11 (16.2%)
Bachelor's degree or higher	1 (1.5%)
**Service location**	
Hospital A	20 (29.4%)
Hospital B	18 (26.5%)
Hospital C	15 (22.1%)
Hospital D	15 (22.1%)

### The results of the GHS sign perception questionnaire

After completing the GHS sign perception questionnaire, people's correct perception level of each sign was determined and compared with the ANSI Z535.3 standard, and the results of its frequency distribution are presented in Table 2. By examining all the presented GHS signs, it was found that among these 11 signs, the participants had a higher correct perception than the ANSI Z535.3 standard in only two of these signs (18.2%), and in nine cases (81.8%) their correct perception was lower than this standard. Among the investigated signs, "Flammable substances" and "Harmful to the environment" signs had the highest, and "Skin irritant" signs had the lowest correct perception (Table 2).

**Table 2:** Distribution of the frequency of GHS signs according to the cleaning workers' correct perception level (*n* = 68, August 2022, Tehran)

Signs	Hospital A			Hospital B			Hospital C			Hospital D			All hospitals		
	N	%	C	N	%	C	N	%	C	N	%	C	N	%	C
Risk of explosion	17	85.0	+	18	100	+	5	33.3	-	8	53.3	-	48	70.6	-
Flammable substances	19	95.0	+	18	100	+	11	73.3	-	12	80.0	-	60	88.2	+
Oxidizing substances	13	65.0	-	16	88.9	+	4	26.7	-	2	13.3	-	35	51.5	-
Gas under pressure	13	65.0	-	4	22.2	-	3	20.0	-	10	66.7	-	26	38.2	-
Corrosive substances	16	80.0	-	13	72.2	-	12	80.0	-	12	80.0	-	53	77.9	-
Toxic substances (acute toxicity)	18	90.0	+	18	100	+	9	60.0	-	8	53.3	-	53	77.9	-
Skin irritant	11	55.0	-	13	72.2	-	1	6.70	-	5	33.3	-	17	25.0	-
Risk to health	17	85.0	+	11	61.1	-	7	46.7	-	11	73.3	-	46	67.6	-
Harmful to the environment	19	95.0	+	16	88.9	+	12	80.0	-	13	86.7	+	60	88.2	+
Biological hazards	12	60.0	-	7	38.9	-	8	53.3	-	11	73.3	-	38	55.9	-
The danger of radioactive materials	18	90.0	+	6	33.3	-	11	73.3	-	12	80.0	-	47	69.1	-

*C* Comparison with ANSI Z535.3 limit (85%), *N* Number, *+* Compliant with the standard, *-* Not compliant with the standard

**Fig. 3:**
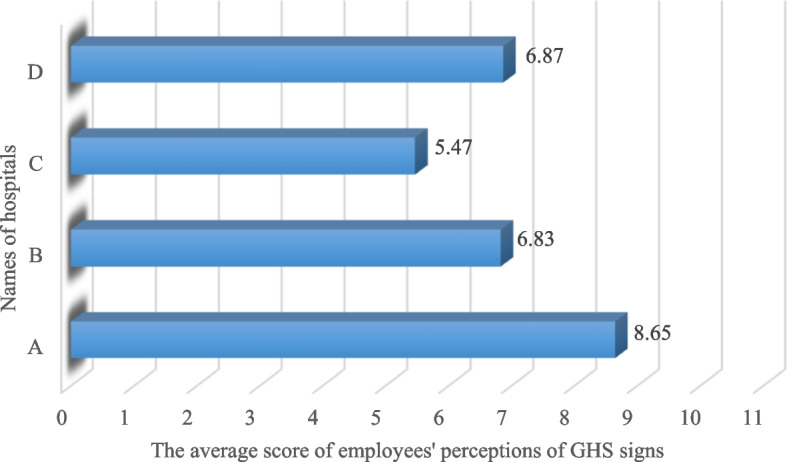
Distribution of cleaning workers' correct perception of GHS signs by hospitals name (*n* = 68, August 2022, Tehran)

Furthermore, 53% and 23.5% of the participants had moderate (3–5), and good (6–8) overall correct perception scores, respectively. Also, the staff of Hospital A had the highest, and the staff of Hospital C had the lowest average score for the overall correct perception of GHS signs. The average scores of the correct perception of cleaning workers of the investigated hospitals were between "Moderate" and "Good" levels and did not reach the "Very good" level (Fig. 3).

### The results of the safety culture questionnaire

**Fig. 4:**
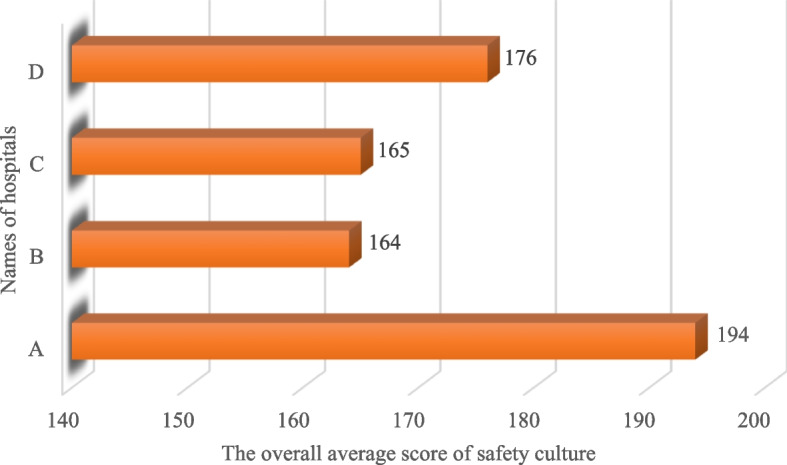
Distribution of the overall average score of cleaning workers' safety culture in the selected hospitals (*n* = 68, August 2022, Tehran)

**Fig. 5:**
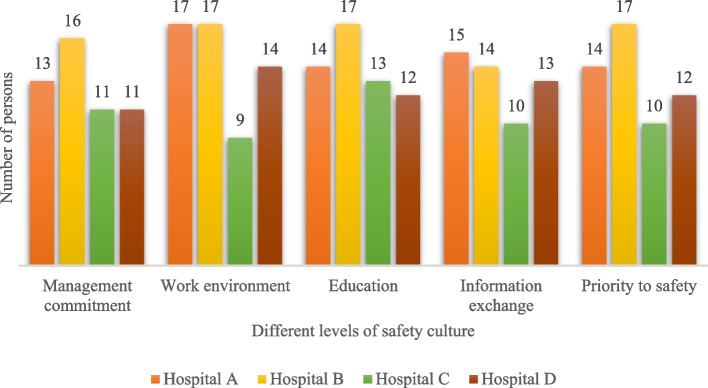
Distribution of participants based on the status of positive attitude towards safety culture (*n* = 68, August 2022, Tehran)

The results of the present study showed that Hospital B had the highest and Hospital C had the lowest overall average score of safety culture (Fig. 4). By examining the cumulative percentage of the safety culture score of the participants, it was also determined that 13 people (19.1%) had an overall negative attitude toward safety culture (< 120), and 55 people (80.9%) had an overall positive attitude toward safety culture (> 120). In addition, among the five investigated levels of safety culture in selected hospitals, the levels of "Work environment" with 83.8% of people (57 people) and "Information exchange" with 76.5% of people (52 people) had the highest and lowest positive scores of safety culture (> 24), respectively (Fig. 5).

In hospitals A, B, C and D, the highest average safety culture score in different levels belonged to the levels of "Work environment", "Safety priority", "Education", and "Work environment", respectively. On the other hand, the lowest average safety culture score at different levels in hospitals A, B, C and D belonged to "Management commitment", "Information exchange", "Information exchange", and "Management commitment", respectively (Table 3). Table 3 shows the distribution of safety culture scores in selected hospitals by different levels of safety culture.

**Table 3:** Distribution of cleaning workers' safety culture scores in the selected hospitals by different levels of safety culture (*n* = 68, August 2022, Tehran)

	Hospital A					Hospital B				
	Education	Work environment	Information exchange	Management commitment	Priority to safety	Education	Work environment	Information exchange	Management commitment	Priority to safety
Minimum	22	20	21	17	21	24	21	20	24	24
Maximum	38	40	39	40	39	34	35	34	39	35
Mean	28.80	29.10	29.05	27.15	28	28.72	29.33	27.94	30.28	29.39
Standard deviation	5.207	5.447	5.326	5.967	5.058	2.845	3.678	3.827	4.548	3.567
	Hospital C					Hospital D				
	Education	Work environment	Information exchange	Management commitment	Priority to safety	Education	Work environment	Information exchange	Management commitment	Priority to safety
Minimum	21	22	21	22	22	23	23	24	20	23
Maximum	36	33	32	33	32	35	37	34	36	37
Mean	27.67	26.27	25.80	26	26.07	28.53	30.27	29.07	27.2	28.13
Standard deviation	3.754	3.712	3.342	3.047	3.035	3.583	5.271	2.939	4.709	4.307

### The relationship between demographic variables and the overall perception of GHS signs and safety culture

A simple linear regression test was used to investigate the relationship between demographic variables and the participants' overall perception of GHS signs and safety culture. According to the findings of this test, only the variables of age and work experience in hospital A had a significant relationship with the overall perception of the GHS signs (Table 4, *P* = 0.029 and *P* = 0.022, respectively).

**Table 4:** Correlation of demographic variables with the cleaning workers' overall perception of GHS signs (*n* = 68, August 2022, Tehran)

		Hospital A				Hospital B			
The first variable	The second variable	β	B	SE	*P*	β	B	SE	*P*
Age	The overall perception	-0.697	-0.191	0.079	0.029*	0.034	0.012	0.173	0.948
Work experience	The overall perception	0.711	0.276	0.107	0.022*	-0.010	-0.003	0.140	0.984
Gender	The overall perception	0.285	1.151	0.895	0.218	0.426	1.019	0.660	0.149
Education	The overall perception	0.377	0.898	0.566	0.135	-0.101	-0.200	0.542	0.719
Marital status	The overall perception	-0.366	-1.540	0.919	0.116	-0.202	-0.483	0.723	0.516
		Hospital C				Hospital D			
The first variable	The second variable	β	B	SE	*P*	β	B	SE	*P*
Age	The overall perception	-0.140	-0.025	0.079	0.755	0.155	0.038	0.100	0.715
Work experience	The overall perception	0.499	0.121	0.120	0.340	-0.173	-0.068	0.167	0.694
Gender	The overall perception	-0.006	-0.014	0.681	0.984	-0.530	-1.717	1.059	0.139
Education	The overall perception	0.490	0.549	0.434	0.238	0.271	0.536	0.617	0.408
Marital status	The overall perception	-0.258	-0.524	0.683	0.463	0.459	1.544	1.372	0.290

*Significance, *SE* Standard Error, *B and SE* unstandardized coefficients, *β* standardized coefficient

### The relationship between safety culture and participants' overall perception of GHS signs

Pearson's correlation test investigated the relationship between safety culture and the participants' overall perception. The obtained results showed a direct and significant relationship between the overall score of safety culture and the overall perception of the signs of GHS (Correlation Coefficient (CC) = 0.313, *P* = 0.009). In addition, there was a significant relationship between the participants' overall perception of GHS signs and "Management commitment" levels in hospitals A (CC = 0.760, *P* =  < 0.001) and C (CC = 0.609, *P* = 0.016), the "Work environment" in hospital C (CC = 0.540, *P* = 0.035), "Education" in hospital A (CC = 0.647, *P* = 0.002), and "Information exchange" in hospitals A (CC = 0.532, *P* = 0.016) and B (CC = 0.725, *P* =  < 0.001) (Table 5).

**Table 5:** Examined correlations between the cleaning workers*'* overall perceptions of GHS signs with the safety culture score (*n* = 68, August 2022, Tehran)

The first variable	The second variable	Hospital A		Hospital B		Hospital C		Hospital D	
		Correlation	*P*	Correlation	*P*	Correlation	*P*	Correlation	*P*
Education	The overall perception of GHS signs	0.647	0.002*	0.173	0.492	-0.014	0.961	0.483	0.068
Work environment	The overall perception of GHS signs	0.136	0.567	-0.243	0.332	0.540	0.038*	0.315	0.254
Information exchange	The overall perception of GHS signs	0.532	0.016*	0.725	<0.001*	0.493	0.062	0.214	0.443
Management commitment	The overall perception of GHS signs	0.760	<0.001*	0.404	0.096	0.609	0.016*	0.234	0.400
Priority to safety	The overall perception of GHS signs	0.103	0.665	0.173	0.492	0.345	0.208	0.273	0.324

*Significance

## Discussion

Healthcare workers are in high-risk occupations and exposed to various potential health risks. Accordingly, it is necessary to improve the perception and safety culture in many fields, including hazardous chemicals, to prevent and deal with these risks. The review of the background of the present study shows that only some studies have paid attention to the issue of perception and safety culture regarding chemical exposures of hospital service workers. Meanwhile, this group of employees working in hospitals plays an essential role in hospitals' quality, order, and cleanliness. This study aimed to investigate the perception of GHS signs and evaluate the safety culture among service workers working in selected hospitals in Tehran.

In general, the results obtained from this study showed that hospital cleaning workers’ correct perception in nine cases (81.8%) of GHS signs was lower than the ANSI Z535.3 standard, which indicates the low familiarity of these people with GHS signs and, as a result, a low correct perception score. These findings were consistent with the study of Jahangiri et al. conducted among chemical industry employees. In their study, all studied GHS signs had a lower score rate than ANSI Z535.3 acceptable limit (< 85%) [19]. It seems that the reason for this alignment is the lack of education regarding the description of chemical hazards according to GHS in the target groups.

However, in a series of other studies, there has been evidence that people were familiar with the signs of chemicals. For example, in a study conducted with the participation of 175 chemistry students, the results showed that most (81%) had a high level of familiarity and perception of the signs of laboratory chemicals. In this research, the students who did not have a correct perception of the signs pointed to reasons such as not paying attention to the labels of chemical substances and problems remembering and understanding more of these signs [7].

The findings of the present study showed that among the GHS signs examined among the service workers of the studied hospitals, the signs "Harmful to the environment" and "Skin irritant" had the highest (88.2%) and the lowest (25%) correct perception among the service workers, respectively. This is while, in a cross-sectional study, the signs of "Flammable substances" (95%) and "Acute toxicity" (94%) had the highest, and "Oxidizing" (7%) and "Compressed gas" (7%) signs had the lowest level of correct perception among cleaning workers [18]. In other studies with the participation of other workers, some signs were more understood, and some were less understood. In the study of Dalvie et al. in South Africa, the highest level of correct perception was related to "Toxic substances" (98%) [34]; in the study of Su and Hsu in Taiwan, the highest level of perception was related to the sign of "Respiratory hazards" (95.8%) [35], in Jahangiri et al.'s study, the highest and lowest levels of correct perception of signs were related to "Acute toxicity" (74.1%), "Explosives", and "Flammable substances"(78.1%) [19]. It seems that there are probably differences in the education style and attitude of people, and the nature of people's jobs in different job groups plays a vital role in the level of correct perception of signs.

Among the hospitals under study, hospitals A and C had the highest and lowest levels of correct perception of GHS signs, respectively, due to reasons such as people's reluctance to learn GHS signs, lack of necessary training, or incomplete and insufficient training in this field among people. Past studies have proven a strong correlation between a lack of knowledge and training in safety signs and labeling and chemical accidents [30, 36–38].

Positive safety culture is considered an influential and critical factor in preventing workplace accidents. The present study's findings showed that the highest and lowest overall safety culture scores were related to hospitals A and B, respectively. The reason can be counted in the level of importance and implementation of the five dimensions of safety culture and the differences in software and hardware in the hospital environment from the safety point of view.

In the current study in two hospitals (A and D), the highest average safety culture score at different levels belonged to the "Work environment" level. On the other hand, the two levels of "Management commitment" and "Information exchange" had the lowest average score of safety culture at different levels in the hospitals. In the study of Shekari et al., who evaluated the safety culture among laboratory personnel in the petrochemical industry, the levels of "Priority to safety" (31.9%) and "Management commitment" (25.2%) had the highest and lowest average scores for safety culture, respectively [22]. In addition, in the study of Sukadarin et al., which investigated the safety culture using seven factors, it was revealed that employees had a positive attitude towards six factors, including "Safety management system and procedure" and "Employee’s involvement" and they had a negative attitude towards one factor (Management commitments) [39]. Thus, it seems that the commitment at management levels to promote the safety culture is still weak, and it should be paid much attention in organizations to increase the safety culture.

Among other results of the present study, it can be said that among the different levels of safety culture, the factors of "Work environment" and "Information exchange" had the highest and lowest scores among the safety culture levels. This study's low score on information exchange can be caused by poor communication between people and occupational health professionals, management, and the education department. Inadequate information and non-optimal use of communication platforms such as virtual space, videos, brochures, educational pamphlets, and discussion sessions to facilitate the exchange of information between people can cause poor communication in the organization.

Of course, it should be kept in mind that any change and modification in the direction of improving the safety culture is a long and time-consuming process, and one should spend time and patience to achieve the goals of increasing a positive safety culture in order to achieve favorable results and feedback. Pearson's correlation test variables showed a significant and positive relationship between the two components of having a positive safety culture and increasing the perception of hospital service staff about GHS signs. This finding shows that the safety culture can increase the perception of safety and the understanding of the danger signs of chemicals and ultimately reduce the risks and accidents related to working with chemicals that employees face daily.

In addition, socio-demographic characteristics can also influence the employees' correct perception of GHS signs. For example, in Jahangiri et al.'s study, employees' perception of GHS symptoms had a significant relationship with variables of gender and education of participants [19]. Also, in our research, the simple linear regression test results showed that age and work experience are two influential factors in the overall perception of employees about GHS signs. With the increase of these factors in hospital A, the employees' perception of GHS symptoms increased. Based on this, it is necessary to pay more attention to socio-demographic factors such as age, work experience, and education and try to make people more familiar with these pictograms by conducting training and retraining courses.

Among the limitations of this study, it was carried out in a survey and cross-sectional manner. In addition, since a questionnaire was used in this research, there is a possibility of bias in the obtained data. For this purpose, it is recommended to other researchers conduct future research longitudinally using other tools in addition to questionnaires and informational data (if the information is provided).

Furthermore, the incomprehensible and Insufficient information of some GHS pictograms for cleaning workers, despite holding training courses in hospitals, was one of the limitations of Standard ANSI Z535.3. In this regard, researchers should have given more explanations about these pictograms. Also, the interference of the time of completing the questionnaires with the working time of the employees and the need for a suitable place to conduct this research and interview with the employees were other limitations of this research.

## Conclusion

Since the cleaning chemicals used in hospitals have adverse effects that can overshadow the public health of people, the hospital cleaning staff must have the necessary knowledge and perceptions about the characteristics and effects of these materials with the appropriate level of safety culture. Thus, in this research, which was based on an analytical-descriptive method, the level of hospital cleaning worker's perception of the signs of chemical substances was evaluated, as well as the evaluation of the safety culture score from the five dimensions of "Education", "Information exchange", "Work environment", "Priority to safety", and "Management commitment". This research showed that the correct perception of hospital cleaning staff was lower than the ANSI Z535.3 standard in 9 out of 11 presented GHS pictograms. Thus, it is necessary to take the necessary measures for the safe training of the high-risk occupational group to increase their perception. On the other hand, in examining the issue of employee safety culture, although the safety culture score of some dimensions, such as "Work environment", was higher than others, such as "Information exchange" and "Management commitment", it should be kept in mind that "Management commitment" and "Information exchange" are not the only influencing factors in safety culture, and other factors are also involved. Therefore, in addition to increasing safety, measures should be taken to increase the score of all safety culture levels.

## Data Availability

The dataset used and analyzed for this study is available from the corresponding author upon reasonable request.

## References

[1] Wang X, Zhang Y, Wu Q, Jin X (2022). Assessing Chemical Safety Knowledge of University Students─ A Case Study. J Chem Educ.

[2] Fayazi A, Pouyakian M, Jafari MJ, Khodakarim S (2019). Development and validation of two awareness and current status assessment questionnaires for the hazardous chemically-exposed staffs though Globally Harmonized System of Classification and Labeling of Chemicals (GHS). Health Saf Work.

[3] Zaip ND, Samad NI, Naim F, Hamzah NA (2021). Assessment of chemical safety awareness among university laboratory workers. Malaysian J Med Health Sci.

[4] Ruokolainen J, Hyttinen M, Sorvari J, Pasanen P (2022). Exposure of cleaning workers to chemical agents and physical conditions in swimming pools and spas. Air Qual Atmos Health.

[5] Ziara KS, Ibraheem AK, Al-Furaiji A (2021). Chemical safety awareness for undergraduate analytical chemistry students: a case study at Baghdad University, Republic of Iraq. Scholars Int J Chem Mater Sci.

[6] Mahmoudi D, Nazari S, Derakhshani M, Dalili A, Hazrati S, Nazari J. The occupational health student’s awareness towards the globally harmonized system for the chemical safety signs. Saf Promot Inj Prev (Tehran). 2018;5(4):219–26.

[7] Mehrifar Y, Eskandarnia A, Pirami H, Mardanparvar H (2016). Assessment of awareness and comprehension of chemical hazard symbols among chemistry students. J Occup Health Epidemiol.

[8] Fayazi A, Pouyakian M, Jafari MJ, Khodakarim S (2020). A survey among three Iranian occupational groups: general knowledge of chemical safety and familiarity with GHS and outdated labeling systems. J Chem Health Saf.

[9] Taş TA, Akiş N, Saricaoğlu H (2021). Occupational Contact Dermatitis in Hospital Cleaning Workers. Dermatitis.

[10] Ecin SM, Sandal A, Çetintepe SP, Koyuncu A, Kurt ÖK, Yıldız AN, et al. Prevalence and Risk Factors of Work-Related Asthma in Hospital Cleaning Workers. Turk Thorac J. 2022;23(3):143–51. 10.5152/TurkThoracJ.2022.21183 PMC9450038 35579226

[11] Attia NM, Ali SA, Ahmed FM (2022). Occupational health hazards among housekeeping workers at Zagazig University Hospitals. Chin J Ind Hyg Occup Dis.

[12] Anza M, Bibiso M, Kuma B, Osuman K (2016). Investigation of laboratory and chemical safety in Wolaita Sodo University. Ethiop Chem Mater Res.

[13] Walters AU, Lawrence W, Jalsa NK (2017). Chemical laboratory safety awareness, attitudes and practices of tertiary students. Saf Sci.

[14] Miller MS. Factors affecting comprehensibility of the globally harmonized system of chemicals in the United States. Carbondale: Southern Illinois University; 2020.

[15] Nayar G. A framework for the sound management of chemicals. Engineering and Physical Sciences Research Council (EPSRC), University of Surrey and Rolls-Royce Ltd. 2020;2:113–27.

[16] Aungkavattana P, Indaraprasirt R, Papan J, Thongkam W, Karlaganis G (2021). The nanosafety and ethics strategic plan of Thailand in the context of the strategic approach to international chemicals management. Toxicol Environ Chem.

[17] Rossete CA, Ribeiro MG (2021). Laboratory technicians’ use and interpretation of hazard communication elements on chemical labels. J Chem Health Saf.

[18] Van der Haar R, Portell M (2015). Comprehension of hazard pictograms of chemical products among cleaning workers. Arch Prev Riesgos Labor.

[19] Jahangiri M, Omidvary F, Maghsoudi A (2018). A Comparison study of perception towards Chemical Hazard Warning Signs in Old and Globally Harmonized System (GHS) among chemical workers in Shiraz. Iran Iran Occupational Health.

[20] Geuens M, Byrne D, Boeije G, Peeters V, Vandecasteele B (2021). Investigating the effectiveness of simplified labels for safe use communication: the case of household detergents. Int J Consum Stud.

[21] Rezaei MS, Golbabaei F, Behzadi MH (2017). Assessing the healthcare workers’ knowledge, attitude, and practice toward health, safety, and environment in an educational hospital affiliated by Iran university of medical sciences (2012–2013). J Environ Sci Technol.

[22] Shekari M, Shirali GA, Hosseinzadeh T (2014). Safety culture assessment among laboratory personnel of a petrochemical company. Health Saf Work.

[23] Arzahan IS, Ismail Z, Yasin SM (2022). Safety culture, safety climate, and safety performance in healthcare facilities: a systematic review. Saf Sci.

[24] Tappura S, Jääskeläinen A, Pirhonen J (2022). Creation of satisfactory safety culture by developing its key dimensions. Saf Sci.

[25] Naji GM, Isha AS, Alazzani A, Saleem MS, Alzoraiki M. Assessing the mediating role of safety communication between safety culture and employees safety performance. Frontiers in Public Health. 2022;10:1–17. 10.3389/fpubh.2022.840281 PMC8960200 35359765

[26] International Nuclear Safety Advisory Group. Summary report on the Post-Accident review meeting on the Chernobyl accident. International Atomic Energy Agency; 1986.

[27] Upadhyay S, Weech-Maldonado R, Lemak CH, Stephenson AL, Smith DG (2021). Hospital staffing patterns and safety culture perceptions: The mediating role of perceived teamwork and perceived handoffs. Health Care Manage Rev.

[28] Tsaur CC, Lee JC (2022). Promoting hospital safety culture: the perspective of safety leadership. Hu Li Za Zhi.

[29] Pimentel MP, Choi S, Fiumara K, Kachalia A, Urman RD (2021). Safety culture in the operating room: variability among perioperative healthcare workers. J Patient Saf.

[30] Davoodi R, Shabestari MM, Takbiri A, Soltanifar A, Sabouri G, Rahmani S (2013). Patient safety culture based on medical staff attitudes in Khorasan Razavi hospitals. Northeastern Iran Iran J Public Health.

[31] Arzahan IS, Ismail Z, Yasin SM, Nordin N, Idris ND, Jusoh JM (2022). A ten-year review: Safety Culture and Safety Performance Studies in Malaysia. Cent Asia Caucasus.

[32] Klaschka U (2012). Dangerous cosmetics-criteria for classification, labelling and packaging (EC 1272/2008) applied to personal care products. Environ Sci Eur.

[33] Mohammadfam I, Mahmoudi Sh (2009). Assessment of HSE culture among MAPNA group’s staffs.

[34] Dalvie MA, Rother HA, London L (2014). Chemical hazard communication comprehensibility in South Africa: Safety implications for the adoption of the globally harmonised system of classification and labelling of chemicals. Saf Sci.

[35] Su TS, Hsu IY (2008). Perception towards chemical labeling for college students in Taiwan using Globally Harmonized System. Saf Sci.

[36] Hill RH (2016). Undergraduates need a safety education!. J Chem Educ.

[37] Omidvari M, Mansouri N, Nouri J (2015). A pattern of fire risk assessment and emergency management in educational center laboratories. Saf Sci.

[38] Abu-Siniyeh A, Al-Shehri SS (2021). Safety in Medical Laboratories: Perception and Practice of University Students and Laboratory Workers. Appl Biosaf.

[39] Sukadarin EH, Suhaimi NS, Abdull N (2012). Preliminary study of the safety culture in a manufacturing industry. Int J Humanit Soc Sci.

